# Supplementary material for: Barium Chemosensors with Dry-Phase Fluorescence for Neutrinoless Double Beta Decay
Source: Sci Rep. 2019 Oct 22;9:15097. doi: 10.1038/s41598-019-49283-x (PMC6805857; doi:10.1038/s41598-019-49283-x)
Supplement: Supplementary file 1 — Supplementary information [file 41598_2019_49283_MOESM1_ESM.pdf]

Supporting Information

# Barium Chemosensors with Dry-Phase Fluorescence for Neutrinoless Double Beta Decay

Pawan Thapa[1,2*,] I. Arnquist[3], N. Byrnes[1], A. A. Denisenko[2], F. W. Foss Jr.[2], B. J. P. Jones[1], A. D. McDonald[1], D. R. Nygren[1], and K. Woodruff[1]

[1]Department of Physics, University of Texas at Arlington, Arlington, TX 76019, USA
[2]Department of Chemistry, University of Texas at Arlington, Arlington, TX 76019, USA
[3]Pacific Northwest National Laboratory (PNNL), Richland, WA 99352, USA
*pawan.thapa@uta.edu

## Table of Contents:


1. **Synthesis of 1-pyrenemethyl bromide (1c) and 9-anthacenemethyl bromide[3] (1d)** **S3**
2. **Synthesis of *N*-protected protected diethanolamine derivatives** **S5**
3. **Synthesis of ethylene glycol derivatives** **S7**
4. **Synthesis of N-methylpyrenyl_monoazacrown ether and N-methylanthracenyl monoazacrown ether** **S8**
5. **NMR experiments of monoaza-18-crown-6 and non-crown (lin) with barium perchlorate in Acetonitrile-$d_3$ at room temperature** **S12**
6. **Critical Micelle Concentration determination** **S13**
7. **NMR figures** **S14**
8. **References** **S22**

## 1. Synthesis of 1-pyrenemethyl bromide (1c)[1,2] and 9-anthacenemethyl bromide[3] (1d)

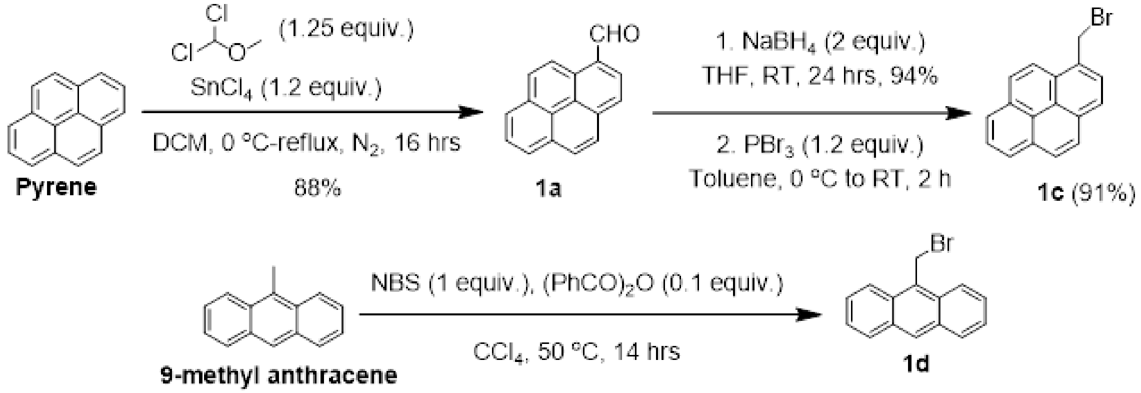


The synthesis of bromomethylpyrene **1c** was accomplished following standard methodology described initially in Ref. 1 and 2. Briefly, pyrene was formylated with dichloromethyl methyl ether as a surrogate for chloroformate, when added to tin tetrachloride. The Friedel-Craft's acylation is achieved in good yields to provide **1a**. Reduction of the aldehyde under standard sodium borohydride conditions followed by PBr3 mediated bromination led to **1c** in 72% overall yield. The 9-bromomethylanthracene **1d** was prepared directly through radical benzylic bromination of 9-methylanthracene with *N*-bromosuccinimide and benzoyl peroxide initiator in one step to give fairly pure **1d**, which is pure enough to be used in subsequent reactions without further purification.

**1-pyrenemethyl carboxaldehyde (1a)**[1]:

Pyrene (2.0 g, 10.0 mmol) was dissolved in 50 mL freshly distilled dichloromethane. The solution was cooled to 0 °C and purged with nitrogen, after which $SnCl_4$ (1.65 mL, 12.0 mmol) was added at once via micro syringe. α,α'-dichloromethyl methyl ether (1.15 mL, 12.5 mmol) was then added dropwise under $N_2$ while maintaining temperature below 5 °C. The resulting mixture was warmed slowly to reflux over 2 hour and further stirred for 16 hours at reflux condition. After the completion of reaction, the reaction mixture was first cooled below 10 °C, diluted with 25 mL dichloromethane and hydrolyzed by carefully adding 50 mL cold water. The immiscible layers were suspended in separatory funnel and the organic phase was separated and dried under $Na_2SO_4$. The crude product was concentrated in rotary evaporator and purified

using column chromatography in silica gel (hexanes: ethyl acetate, 10:1) to obtain yellow solid Pyrene-4-carbaldehyde as desired product in 88% yield.

Yield: 2.02 g, (88%); **$^1$H NMR** (500 MHz, $CDCl_3$) δ 10.72 (s, 1H), 9.34 (d, *J* = 9.3 Hz, 1H), 8.36 (d, *J* = 7.9 Hz, 1H), 8.28 – 8.21 (m, 3H), 8.20 – 8.13 (m, 2H), 8.09 – 7.98 (m, 2H); **$^{13}$C NMR** (125 MHz, $CDCl_3$) δ 193.1, 135.5, 131.4, 131.0, 130.9, 130.8, 130.7, 130.4, 127.3, 127.2, 127.1, 126.9, 126.6, 124.6, 124.5, 124.0, 123.0.

**1-pyrenemethanol (1b)[2]:** $NaBH_4$ (152 mg, 4.0 mmol) was added in small portion into the solution of 1-pyrenecarboxaldehyde **1a** (461 mg, 2.0 mmol) in dry THF (10 mL) at room temperature. The reaction mixture was stirred for 16 hours after which a few drops (2-3 drops) of acetic acid were added to quench the excess $NaBH_4$. The clear solution was concentrated in rotary evaporator. The resulting solid was dissolved in 15 mL dichloromethane and washed with 5 mL water twice. The washed organic solution was dried with anhydrous sodium sulfate and was concentrated to give pale-yellow solid 1-pyrenemethanol as desired product.

Yield: 441 mg (94%); **mp** 122-124 °C; **$^1$H NMR** (500 MHz, $CDCl_3$) δ 8.24 (d, *J* = 9.2 Hz, 1H), 8.16 (d, *J* = 7.3 Hz, 2H), 8.07 – 7.92 (m, 6H), 5.29 (s, 2H); **$^{13}$C NMR** (125 MHz, $CDCl_3$) δ 133.7, 131.2, 131.2, 130.8, 128.7, 127.8, 127.4, 127.4, 126.0, 125.9, 125.3, 125.3, 124.9, 124.7, 124.7, 122.9, 63.7.

**1-pyrenemethyl bromide (1c)[2]: 1b** (100 mg, 0.43 mmol) was dissolved in 5 mL freshly distilled toluene and the reaction vial was sealed properly with rubber septum. $PBr_3$ (49 µL, 0.517 mmol) was added dropwise over 5 minutes at 0 °C and the reaction mixture was stirred for 1 hour at 0 °C and was slowly brought to room temperature. 2 mL saturated $Na_2CO_3$ was added slowly. The crude product was extracted using 5 mL dichloromethane twice. The combined organic phase was dried under anhydrous $Na_2SO_4$ and concentrated to obtain pale yellow solid. The crude solid was recrystallized in methanol to give pale yellow solid 1-pyrenemethyl bromide (**1c**) as desired product.

Yield: 115 mg (91%); **mp** 138-140 °C; **$^1$H NMR** (500 MHz, $CDCl_3$) δ 8.36 (d, *J* = 9.2 Hz, 1H), 8.22 (t, *J* = 7.0 Hz, 2H), 8.20 (d, *J* = 3.4 Hz, 1H), 8.12 – 7.98 (m, 5H), 5.24 (s, 2H); **$^{13}$C NMR** (125 MHz, $CDCl_3$) δ

132.0, 131.3, 130.8, 130.6, 129.1, 128.3, 128.1, 127.8, 127.4, 126.3, 125.7, 125.7, 125.2, 124.9, 124.7, 122.9, 32.3; **IR (neat):** 3035, 2988, 2954, 1594.

**9-anthracenemethyl bromide (1d)[3]:** A solution of 9-methyl anthracene (577 mg, 3.0 mmol), NBS (534 mg, 3 mmol) and benzoyl peroxide (75 mg, 0.3 mmol) in 10 mL $CCl_4$ was stirred for 14 hours at 50 °C. Solution was cooled to room temperature and undissolved solid was filtered and washed with 5 mL diethyl ether. Crude solid product was used as it is for the next step without further purification.

2. **Synthesis of *N*-protected protected diethanolamine derivatives[4]:**

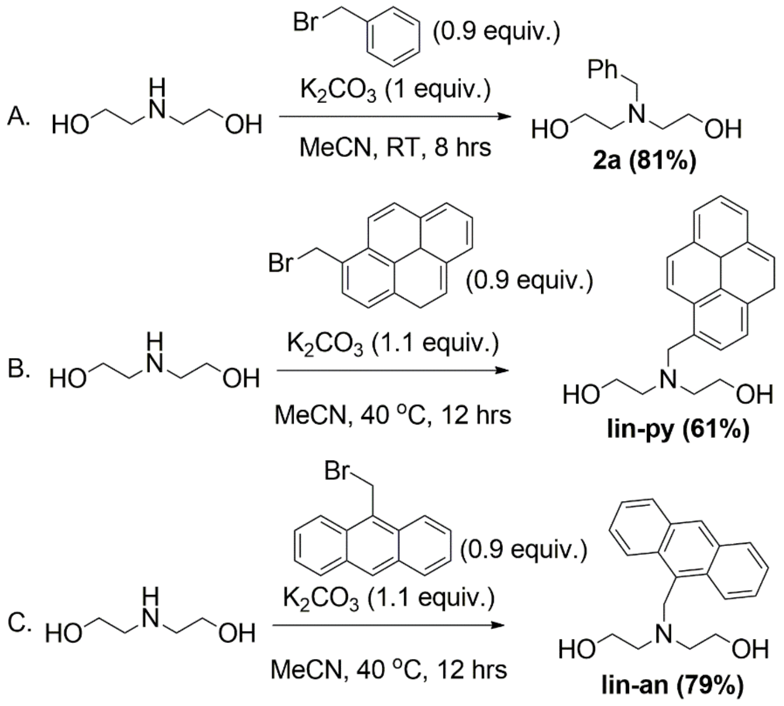


During the construction of the aza-crown ether systems, we found that better overall yields were obtained by the selective *N*-protection of diethanolamine. Benzyl protection proved more fruitful than N-Boc, based on our comparative studies[3]. The standard N-benzyl protection strategy was carried out with inorganic potassium carboxylate in acetonitrile to produce N-benzyl diethanolamine in good yield. Fortuitously, this approach was effective in producing N-pyrenylmethyldiethanolamine (**lin-py**) and N-anthracenylmethyldiethanolamine (**lin-an**) at slightly elevated temperatures, which were used as acyclic controls for the wet and dry studies in our report.

**2,2'-(benzylazanediyl)diethanol (2a):** Benzyl bromide (3 mL, 25 mmol) was added dropwise into the solution of diethanolamine (2 g, 20 mmol) and potassium carbonate (3.4 g, 25 mmol) in 15 mL acetonitrile over 15 minutes at room temperature. The resulting solution was stirred for 14 hours at room temperature. Undissolved solid particles were filtered and washed with 5 mL acetonitrile. Filtrate was concentrated under rotary evaporator under reduced pressure. Crude product was purified in silica gel chromatography using hexanes and ethyl acetate in 1:1 ratio to obtain desired product in 81% yield as colorless liquid.

Yield 3.15 g, 81%; Clear Liquid; **$^{1}$H NMR** (500 MHz, $CDCl_3$) δ 7.34 – 7.12 (m, 5H), 3.66 (s, 2H), 3.56 (t, *J* = 5.4 Hz, 4H), 2.65 (t, *J* = 5.4 Hz, 4H); **$^{13}$C NMR** (125 MHz, $CDCl_3$) δ 138.7, 128.9, 128.3, 127.1, 59.5, 59.1, 55.7.

**2,2'-((pyren-1-ylmethyl)azanediyl)diethanol (lin-py):**

**1c** or **1d** (0.9 mmol) was added into the solution of diethanolamine (105 mg, 1.0 mmol) and potassium carbonate (152 mg, 1.1 mmol) in 5 mL acetonitrile. The resulting solution was stirred for 14 hours at 40 °C. The solution was diluted with 5 mL acetonitrile and the undissolved solid particles were filtered and washed with 5 mL acetonitrile. Filtrate was concentrated under rotary evaporator under reduced pressure. Crude product was purified in silica gel chromatography using hexanes and ethyl acetate in 1:1 ratio to obtain desired product.

Yield 61%, 176 mg; Pale yellow solid; M. Pt. 92 °C (decomp.); **$^{1}$H NMR** (500 MHz, $CDCl_3$) δ 8.48 (d, *J* = 9.2 Hz, 1H), 8.18 (d, *J* = 7.6 Hz, 2H), 8.13 (dd, *J* = 10.9, 8.6 Hz, 2H), 8.06 – 7.95 (m, 4H), 4.39 (s, 2H), 3.57 (t, *J* = 5.3 Hz, 4H), 2.81 (t, *J* = 5.3 Hz, 4H); **$^{13}$C NMR** (125 MHz, $CDCl_3$) δ 132.1, 131.4, 131.1, 130.9, 129.8, 128.4, 127.9, 127.5, 127.4, 126.1, 125.3, 125.2, 124.9, 124.6, 123.3, , 59.9, 58.5, 56.2; **IR** (neat, $cm^{-1}$): 3253 (br), 3045, 2941, 2850, 1139; **HRMS** (ESI) calcd for $C_{21}H_{22}NO_2$ $[M+H]^+$, 320.1645 found, 320.1648

**2,2'-((anthracen-9-ylmethyl)azanediyl)diethanol (lin-an)**

**lin-an** was synthesized following a general reaction condition like **lin-py**.

Yield 79%, 210 mg, Yellow solid; M. Pt. 85 °C (decomp.); **$^1$H NMR** (500 MHz, $CDCl_3$) δ 8.47 (s, 1H), 8.45 (d, *J* = 4.5 Hz, 2H), 8.02 (d, *J* = 8.4 Hz, 2H), 7.59 – 7.51 (m, 2H), 7.52 – 7.43 (m, 2H), 4.72 (s, 2H), 3.54 (t, *J* = 5.3 Hz, 4H), 2.82 (t, *J* = 5.3 Hz, 4H); **$^{13}$C NMR** (125 MHz, $CDCl_3$) δ 131.5, 131.3, 129.5, 128.2, 126.3, 125.0, 124.3, 59.9, 56.0, 51.7; **IR** (neat, $cm^{-1}$): 3220 (br), 3058, 2950, 2858, 1125; **HRMS** (ESI) calcd for $C_{19}H_{22}NO_2$ $[M+H]^+$, 296.1645 found, 296.1644

### 3. Synthesis of ethylene glycol derivatives[5]:

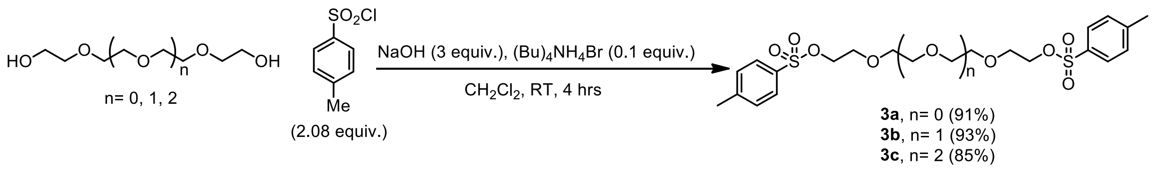


Terminally activated polyethylene glycol derivatives were prepared for cyclization with **2a** by known methods[4]. Dimeric, trimeric, and tetrameric diols were reacted with toluenesulfonyl chloride under basic and phase transfer conditions to prepare the ditosylated crown ether fragments **3a-c** in excellent yields.

**General Procedure for synthesis of ethylene glycol bis-p-toluenesulfonate**: A solution of p-toluenesulfonyl chloride (3.96 g, 20.8 mmol) in 10 mL $CH_2Cl_2$ was added dropwise into the mixture of NaOH (1.2 g, 30.0 mmol), diol (10.0 mmol) and tetrabutylammonium bromide (1.0 mmol) in 20 mL of water and $CH_2Cl_2$ (1:1) over 15 minutes at room temperature. The resulting mixture was stirred vigorously for 4 hours at room temperature. Organic phase was separated and washed with 5 mL water (X3). The combined organic phase was dried under anhydrous $Na_2SO_4$. The crude product was concentrated in rotary evaporator in reduced pressure and finally dried under high vacuum. Crude product was used as it is, and no further purification was carried out.

In case of **2a**, the crude product was obtained as white solid which was purified by recrystallization in methanol to obtain the desired product as white crystalline solid.

**Triethylene glycol bis-p-toluenesulfonate (3a):**

91% yield; White crystalline solid; **$^1$H NMR** (500 MHz, $CDCl_3$) δ 7.78 (d, *J* = 8.2 Hz, 4H), 7.33 (d, *J* = 8.1 Hz, 4H), 4.16 – 4.08 (m, 4H), 3.66 – 3.61 (m, 4H), 3.51 (s, 4H), 2.43 (s, 6H); **$^{13}$C NMR** (125 MHz, $CDCl_3$) δ 144.9, 133.0, 129.9, 128.0, 70.7, 69.3, 68.8, 21.7.

**Tetraethylene glycol bis-p-toluenesulfonate (3b):**

Clear Liquid; $^{1}$**H NMR** (500 MHz, $CDCl_3$) δ 7.74 (d, *J* = 8.3 Hz, 4H), 7.30 (dd, *J* = 8.3, 4H), 4.20 – 3.92 (m, 4H), 3.63 (m, 4H), 3.53 – 3.46 (m, 8H), 2.40 (s, 6H); $^{13}$**C NMR** (125 MHz, $CDCl_3$) δ 144.9, 132.8, 129.9, 127.8, 70.5, 70.3, 69.4, 68.5, 21.5.

**Pentaethylene glycol bis-p-toluenesulfonate (3c):**

Clear Liquid; $^{1}$**H NMR** (500 MHz, $CDCl_3$) δ 7.72 (d, *J* = 8.2 Hz, 4H), 7.28 (d, *J* = 8.1 Hz, 4H), 4.21 – 4.02 (m, 4H), 3.63 – 3.59 (m, 4H), 3.55 – 3.48 (m, 12H), 2.38 (s, 6H); $^{13}$**C NMR** (125 MHz, $CDCl_3$) δ 144.8, 132.8, 129.8, 127.8, 70.6, 70.4, 70.3, 69.2, 68.5, 21.5.

4. **Synthesis of N_methylpyrenyl_monoazacrown ether[5, 6] (15c5-py, 18c6-py, and 21c7-py) and N-methylanthracenyl monoazacrown ether (15c5-an, 18c6-an, and 21c7-an)[7]:**

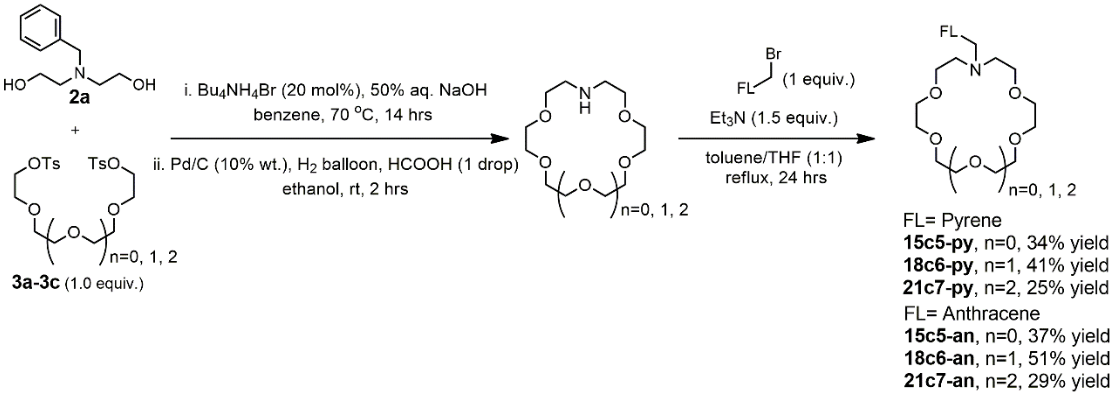


Protected diethanolamine **2a** and activated ethylene glycol fragments **3a-c** were converted to their respective aza-crown ethers in parallel synthesis under elegant and well described phase transfer conditions in a water/benzene mixture at elevated temperatures. The *N*-benzyl-aza-crown ethers were then deprotected by hydrogenolysis under standard conditions at room temperature and slightly elevated (balloon) pressure. The purified aza-crown ethers were then covalently linked to pyrene (**py**, **1c**) or anthracene (**an**, **1d**) fluorophores via $S_N2$ reactions under basic and mixed solvent conditions to provide the desired products used in this study in synthetically useful overall yields (29-51% over three steps)[4-6].

N-benzyldiethanoamine (**2a**) (195 mg, 1.0 mmol), tetrabutylammonium bromide (0.2 mmol) were mixed in 2 mL 50% NaOH solution. Solution of ethylene glycol bis-p-toluenesulfonate (**3a** or **3b** or **3c**) (1.0 mmol) in 12 mL benzene was then added. The resulting mixture was stirred vigorously at 70 °C for 14 hours. The solution was cooled, diluted with 2 mL benzene and 2 mL water and transferred in separatory funnel. The organic phase was separated, washed with water (3 X 4 mL), dried under anhydrous Magnesium sulfate and concentrated under reduced pressure. The crude product was extracted from the residue by washing with boiling hexanes (3 X 5 mL). The combined hexane wash was evaporated under reduced pressure to obtain clear viscous liquid as crude product. No further purification was carried out and the crude product was directly used for the next step reactions.

**N.B**. During the synthesis of **Aza-21-crown-7**, Cesium chloride (3 equiv.) was used as template to facilitate the cyclization reaction in first step. In case of **Aza-18-crown-6**, addition of $BaBr_2$ (as described in literature) as template did not provide improved yield. Therefore, **Aza-15-crown-5** and **Aza-18-crown-6** were synthesized in the absence of templates.

A solution of crude N-benzyl diethanolamine **2a** (100 mg), 10% Pd/C and 1 drop formic acid in 5 mL ethanol was stirred under hydrogen atmosphere ($H_2$ balloon) at room temperature for 2 hours. The catalyst was filtered under celite and washed with 5 mL ethanol. The filtrate was concentrated in rotary evaporator under reduced pressure to obtain clear liquid aza-crown ether product in crude form which was used directly for the next step.

Crude aza-crown ether (50 mg) was added into solution of triethyl amine (1.5 equiv.) in 4 mL toluene and tetrahydrofuran (1:1) and the resulting solution was stirred under Nitrogen for 10 minutes. Bromomethyl pyrene or bromomethyl anthracene (1 equiv. with respect to crude aza- crown ether) was then added in small portion. The mixture was refluxed under stirring for 24 hours at 70 °C under Nitrogen. The reaction mixture was dried under reduced pressure and purified with alumina chromatography using gradient mobile phase from 5:1 hexanes/ethyl acetate to 1:1 hexanes/ethyl acetate to obtain the desired **1-(Pyren-1-ylmethyl)-aza-crown** (**15c5-py, 18c6-py and 21c7-py**) or **1-**

**(Anthracen-9-ylmethyl)-aza-crown** (**15c5-an, 18c6-an and 21c7-an**). Each product was further purified in alumina column using 1% TEA in 1:1 hexanes and ethyl acetate prior to fluorescence studies.

**1-(Pyren-1-ylmethyl)-aza-15-crown-5 (15c5-py):**

Yield: 34 % over three steps; Pale yellow viscous liquid; **$^1$H NMR** (500 MHz, $CDCl_3$) δ 8.57 (d, *J* = 9.2 Hz, 1H), 8.17 (t, *J* = 7.8 Hz, 2H), 8.11 (dd, *J* = 8.4, 5.3 Hz, 2H), 8.06 – 7.98 (m, 4H), 4.37 (s, 2H), 3.74 – 3.60 (m, 16H), 2.94 (t, *J* = 5.9 Hz, 4H); **$^{13}$C NMR** (125 MHz, $CDCl_3$) δ 131.4, 131.0, 130.7, 128.1, 127.5, 127.1, 127.0, 125.8, 125.0, 125.0, 124.9, 124.5, 124.3, 71.1, 70.7, 70.2, 70.1, 59.3, 54.7; **HRMS** (ESI) calcd for $C_{27}H_{32}NO_4$ $[M+H]^+$, 434.2326 found, 434.2315

**1-(Pyren-1-ylmethyl)-aza-18-crown-6 (18c6-py):**

Yield: 41% over three steps; Pale yellow viscous liquid; **$^1$H NMR** (500 MHz, $CD_3CN$) δ 8.64 (d, *J* = 9.3 Hz, 1H), 8.27 – 8.20 (m, 2H), 8.20 – 8.14 (m, 2H), 8.12 – 8.01 (m, 4H), 4.31 (s, 2H), 3.61 – 3.48 (m, 20H), 2.81 (t, *J* = 5.7 Hz, 4H); **$^{13}$C NMR** (125 MHz, $CDCl_3$) δ 132.1, 131.4, 131.2, 129.8, 128.4, 127.9, 127.5, 126.1, 125.3, 124.6, 123.3, 70.9, 70.8, 70.7, 70.6, 60.0, 56.3; **HRMS** (ESI) calcd for $C_{29}H_{36}NO_5$ $[M+H]^+$, 478.2588 found, 478.2581

**1-(Pyren-1-ylmethyl)-aza-21-crown-7 (21c7-py):**

Yield: 25% over three steps; Pale yellow viscous liquid; **$^1$H NMR** (500 MHz, $CDCl_3$) δ 8.62 (d, *J* = 9.0 Hz, 1H), 8.21 – 7.97 (m, 9H), 4.39 (s, 2H), 3.74 – 3.56 (m, 24H), 2.91 (t, *J* = 4.8 Hz, 4H); **HRMS** (ESI) calcd for $C_{31}H_{40}NO_6$ $[M+H]^+$, 522.2850 found, 522.2842

**1-(Anthracen-9-ylmethyl)-aza-15-crown-5 (15c5-an):**

Yield: 37%% over three steps; yellow viscous liquid; **$^1$H NMR** (500 MHz, $CDCl_3$) δ 8.54 (d, *J* = 8.8 Hz, 2H), 8.40 (s, 1H), 7.98 (d, *J* = 8.8 Hz, 2H), 7.54 – 7.41 (m, 4H), 4.61 (s, 2H), 3.70 – 3.65 (m, 8H), 3.65 – 3.57 (m, 8H), 2.92 (t, *J* = 6.0 Hz, 4H); **$^{13}$C NMR** (125 MHz, $CDCl_3$) δ 131.5, 131.4, 130.5, 129.0, 127.5, 125.6, 125.3, 124.8, 71.0, 70.7, 70.1, 70.1, 54.2, 52.6; **HRMS** (ESI) calcd for $C_{25}H_{31}NO_5Na$ $[M+Na]^+$, 432.2145 found, 432.2140

**1-(Anthracen-9-ylmethyl)-aza-18-crown-6 (18c6-an):**

Yield: 51% over three steps; yellow viscous liquid; **$^{1}$H NMR** (500 MHz, $CDCl_3$) δ 8.56 (d, *J* = 8.9 Hz, 2H), 8.39 (s, 1H), 7.98 (d, *J* = 8.9 Hz, 2H), 7.56 – 7.37 (m, 4H), 4.60 (s, 2H), 3.84 – 3.45 (m, 20H), 2.89 (t, *J* = 5.2 Hz, 4H); $^{13}$C NMR (125 MHz, $CD_3CN$) δ 132.3, 132.1, 129.7, 128.7, 128.2, 126.5, 126.3, 125.9, 71.3, 71.1, 70.8, 70.4, 54.7, 52.2; **HRMS** (ESI) calcd for $C_{27}H_{36}NO_5$ $[M+H]^+$, 454.2588 found, 454.2593

**1-(Anthracen-9-ylmethyl)-aza-21-crown-7 (21c7-an):**

Yield: 29% over three steps; yellow viscous liquid; **$^{1}$H NMR** (300 MHz, $CDCl_3$) δ 8.58 (d, *J* = 8.5 Hz, 2H), 8.40 (s, 1H), 7.99 (d, *J* = 8.5 Hz, 2H), 7.57 – 7.37 (m, 4H), 4.62 (s, 2H), 3.78 – 3.46 (m, 24H), 2.88 (t, *J* = 5.4 Hz, 2H); **$^{13}$C NMR** (125 MHz, $CDCl_3$) δ 133.6, 131.5, 130.2, 129.0, 128.5, 125.7, 125.4, 124.9, 71.0, 71.0, 71.0, 70.6, 53.9, 51.8; **HRMS** (ESI) calcd for $C_{29}H_{40}NO_6$ $[M+H]^+$, 498.2850 found, 498.2849

## 5. NMR experiments of monoaza-18-crown-6 and non-crown (lin) with barium perchlorate in Acetonitrile-$d_3$ at room temperature:

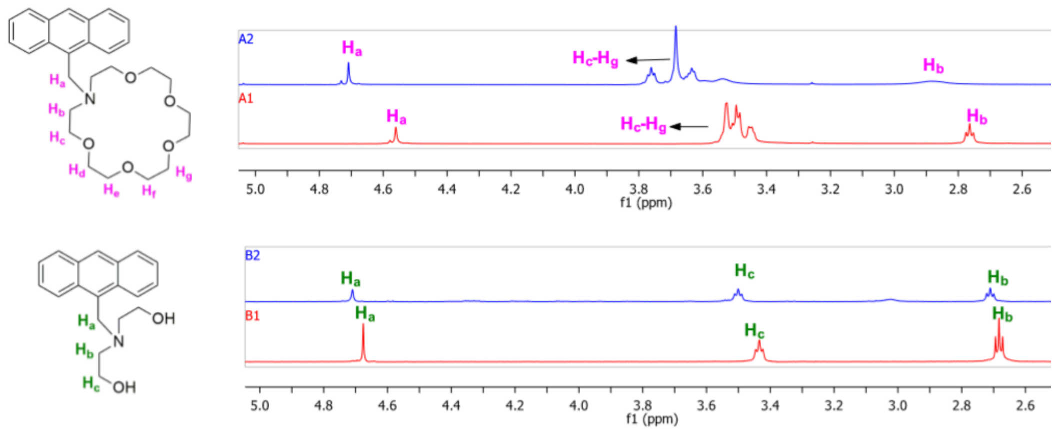


**Figure S1:** Partial $^1$H NMR (500 MHz, 25 °C, acetonitrile-$d_3$) spectra of (A1) **18c6-an** (14 mM) alone without incubation; (A2) **18c6-an** (14 mM) + **$Ba(ClO_4)_2$** (14 mM) after 30 minute incubation; (B1) **lin-an** (14 mM) alone without incubation; (B2) **lin-an** (14mM) + **$Ba(ClO_4)_2$** (14 mM) after 30 minute incubation

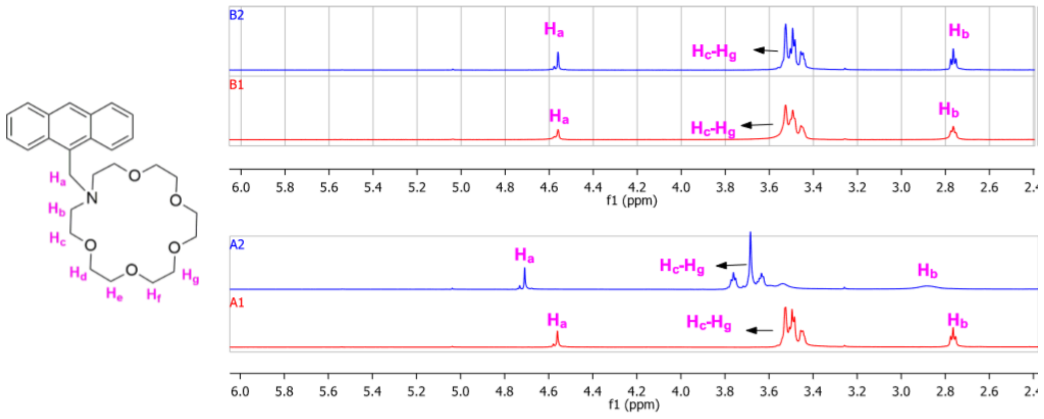


**Figure S2:** Partial $^1$H NMR (500 MHz, 25 °C, acetonitrile-$d_3$) spectra of (A1) **18c6-an** (14 mM) alone without incubation; (A2) **18c6-an** (14mM) + **$Ba(ClO_4)_2$** (14 mM) after 30 minute incubation; (B1) **18c6-an** (14 mM) alone without incubation; (B2) **18c6-an** (14mM) alone after 30 minute incubation

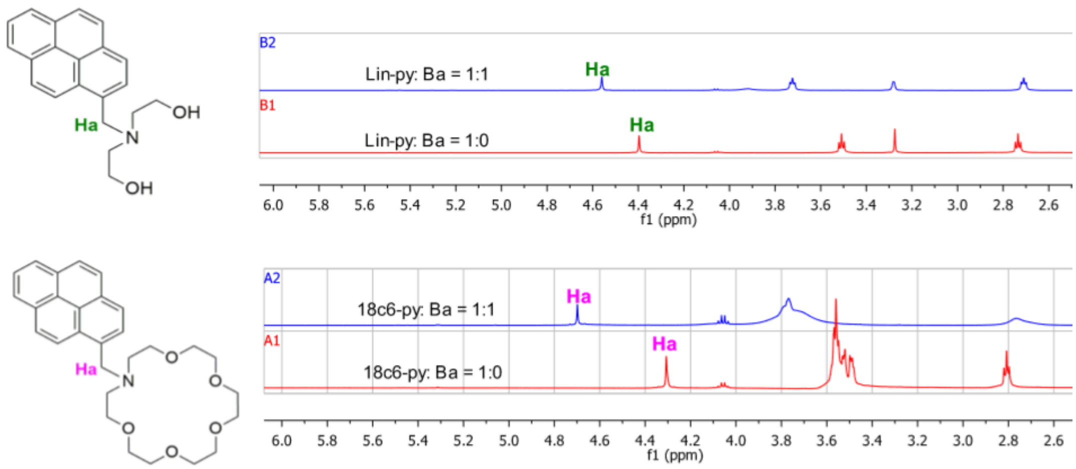


**Figure S3:** Partial $^{1}$H NMR (500 MHz, 25 °C, acetonitrile-$d_3$) spectra of (A1) **18c6-py** (14 mM) alone without incubation; (A2) **18c6-py** (14mM) + **Ba(ClO$_4$)$_2$** (14 mM) after 30 minute incubation; (B1) **lin-py** (14 mM) alone without incubation; (B2) **lin-py** (14mM) + **Ba(ClO$_4$)$_2$** (14 mM) after 30 minute incubation

## 6. Critical Micelle Concentration study with 18c6-py:

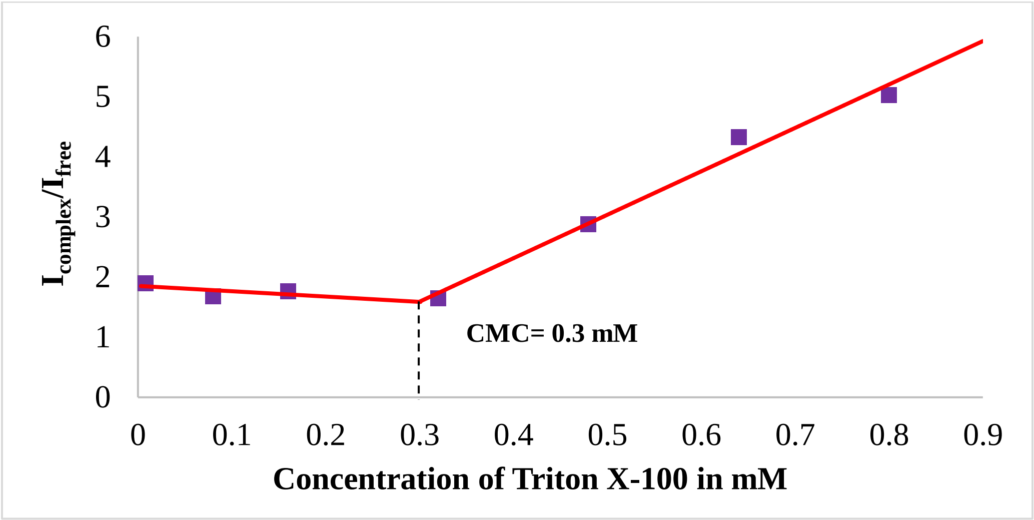


**Figure S4**: Change in the fluorescence intensity of **18c6-py** (2.1 µM) containing **Ba(ClO$_4$)$_2$** (7.5 mM) with different concentration of **Triton X-100** in Tris buffer solution (20 mM, pH = 10.1)

## 7. NMR figures:

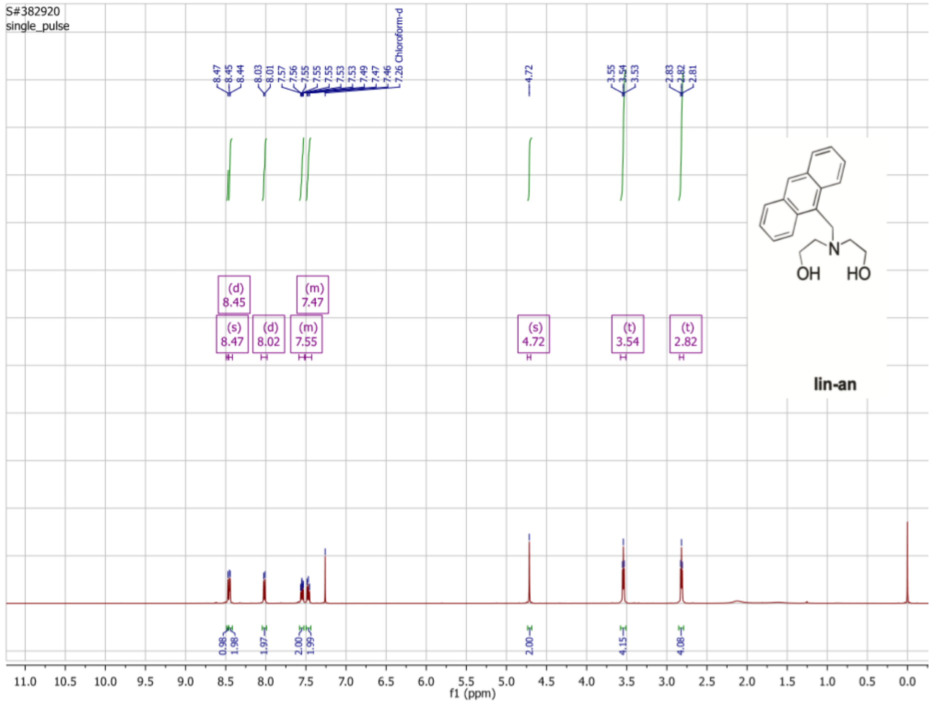


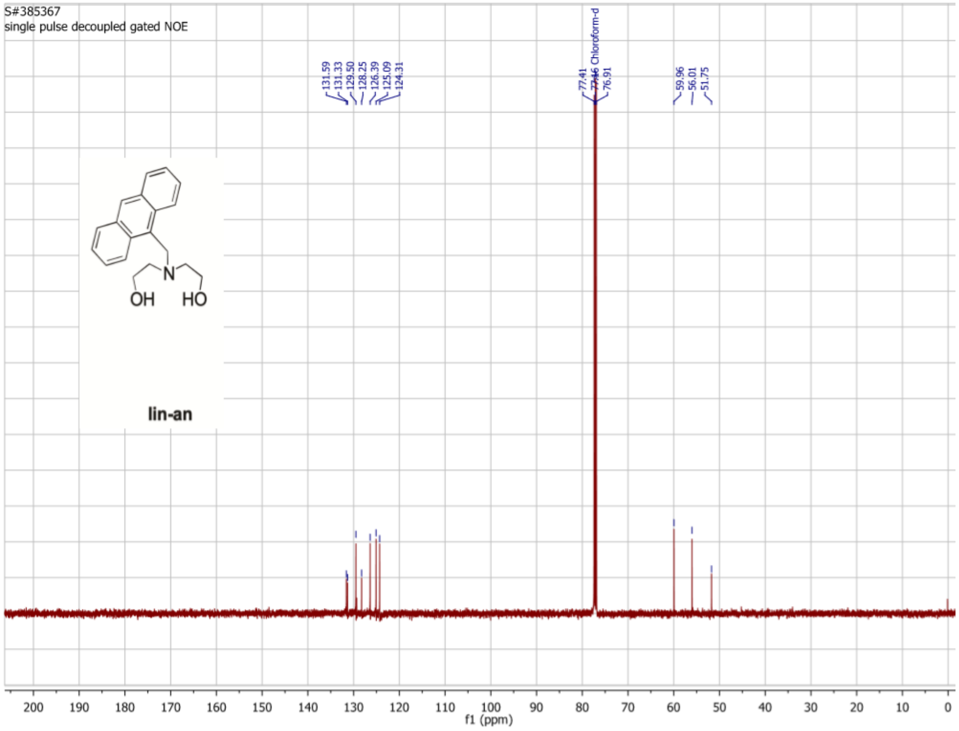


**Figure S5**: $^{1}$H NMR and $^{13}$C NMR of **Lin-an**

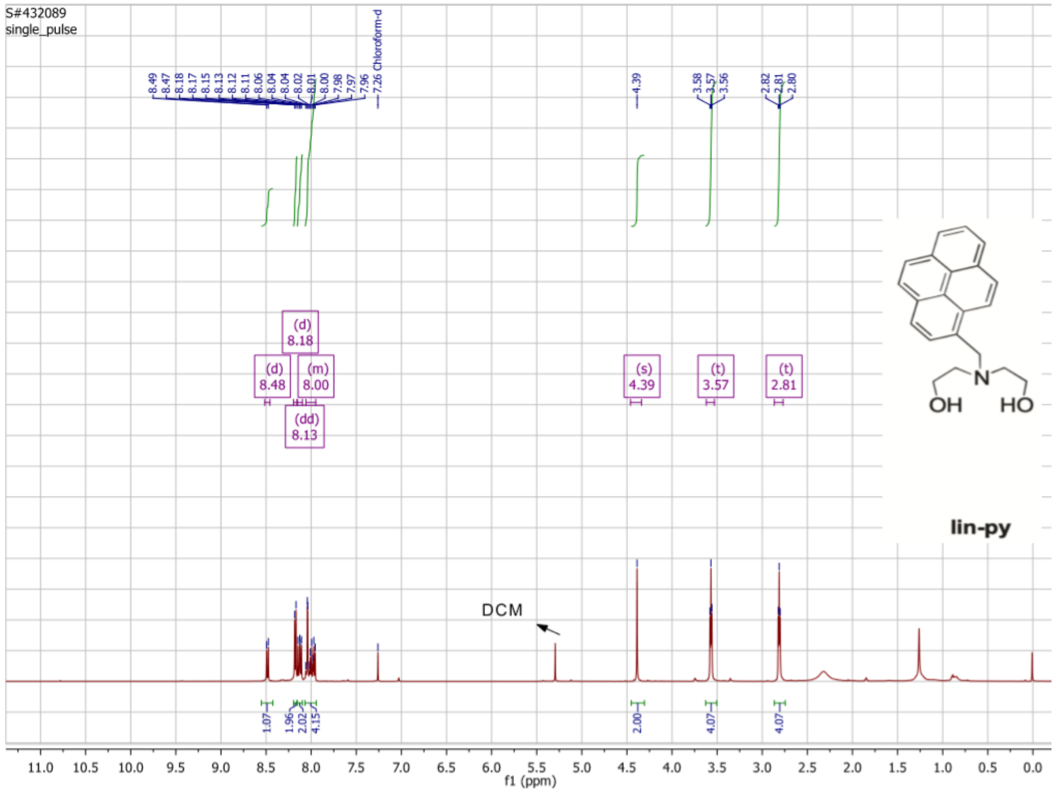


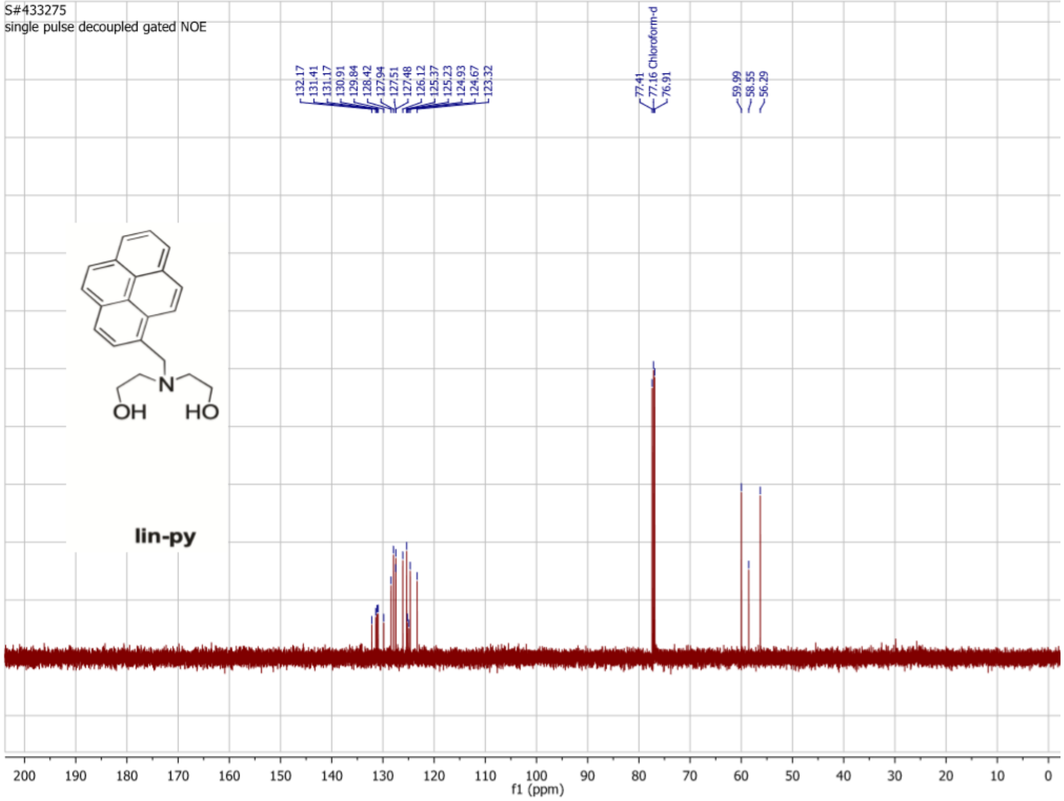


**Figure S6**: $^{1}$H NMR and $^{13}$C NMR of **Lin-py**

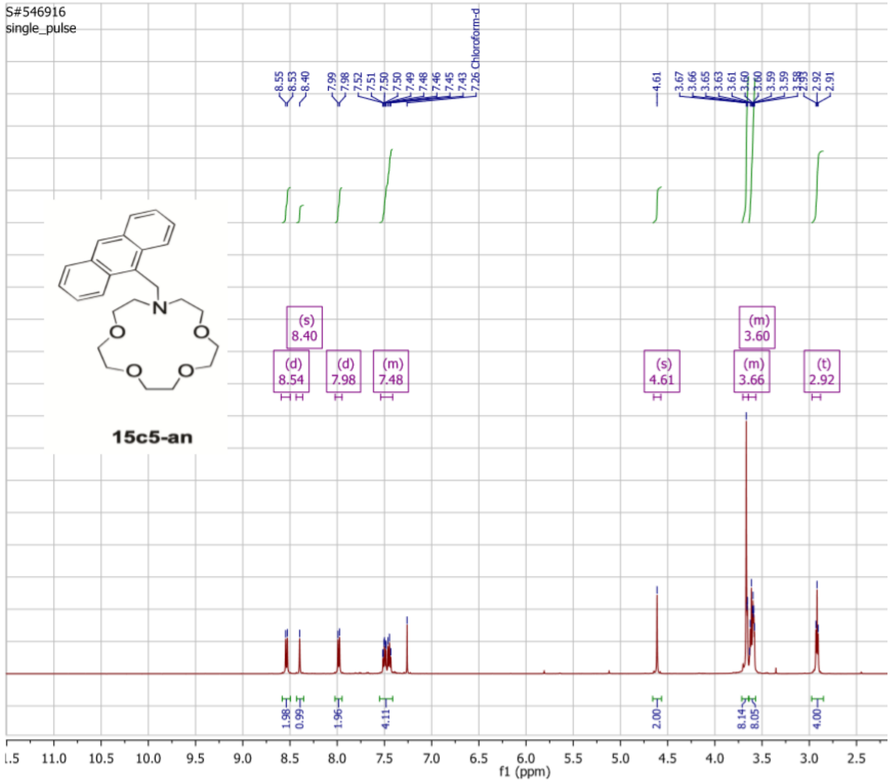

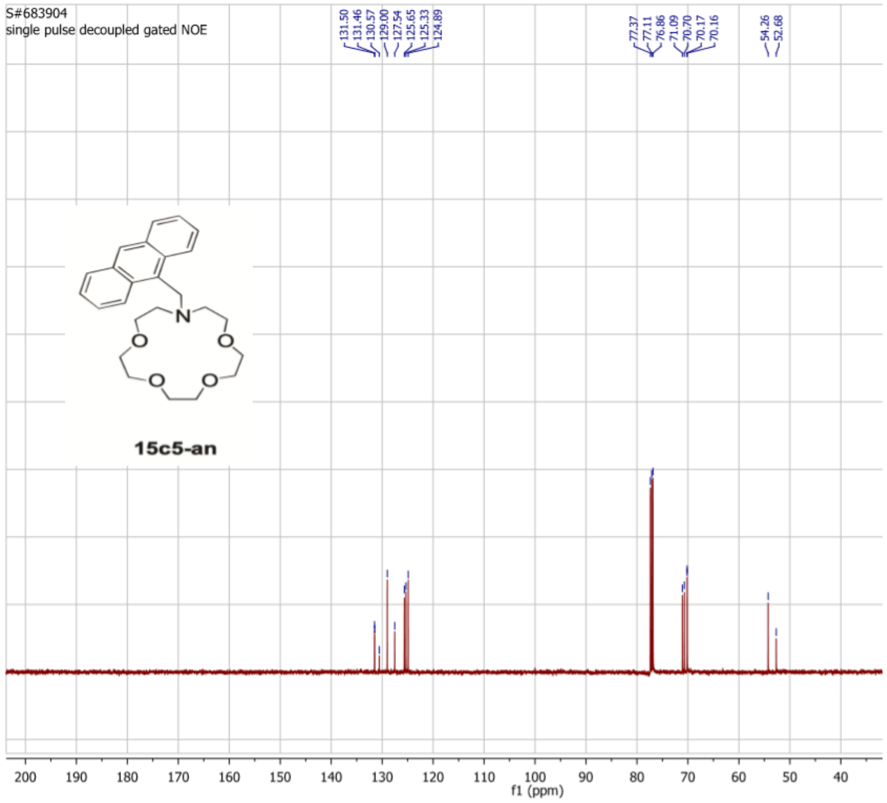

**Figure S7**: $^{1}$H NMR and $^{13}$C NMR of **15c5-an**

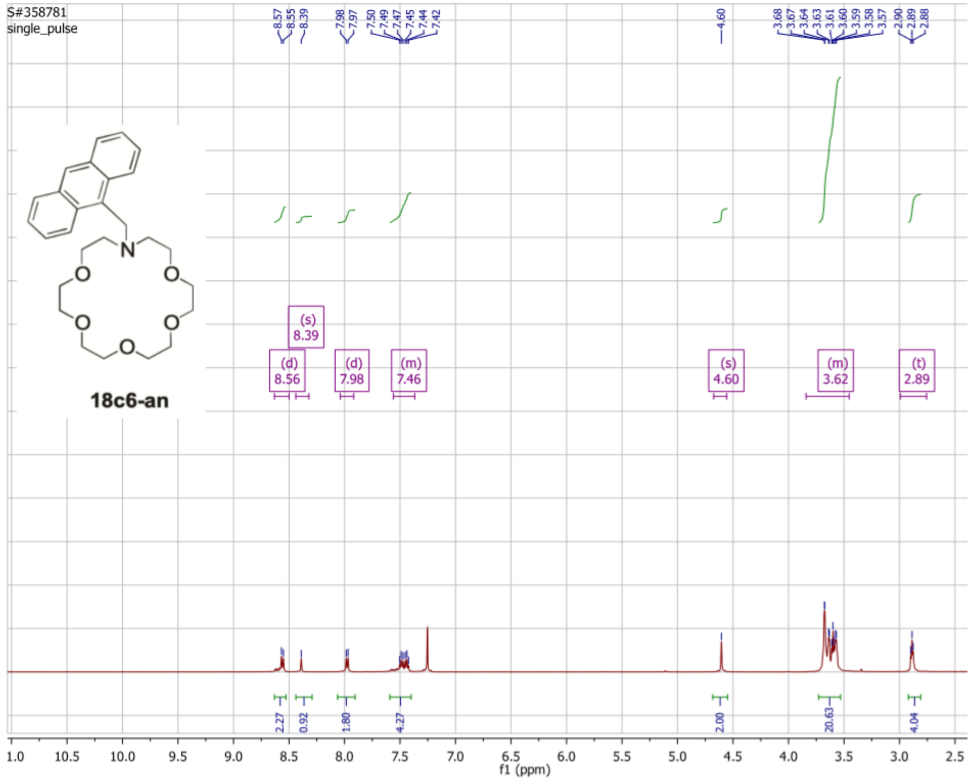

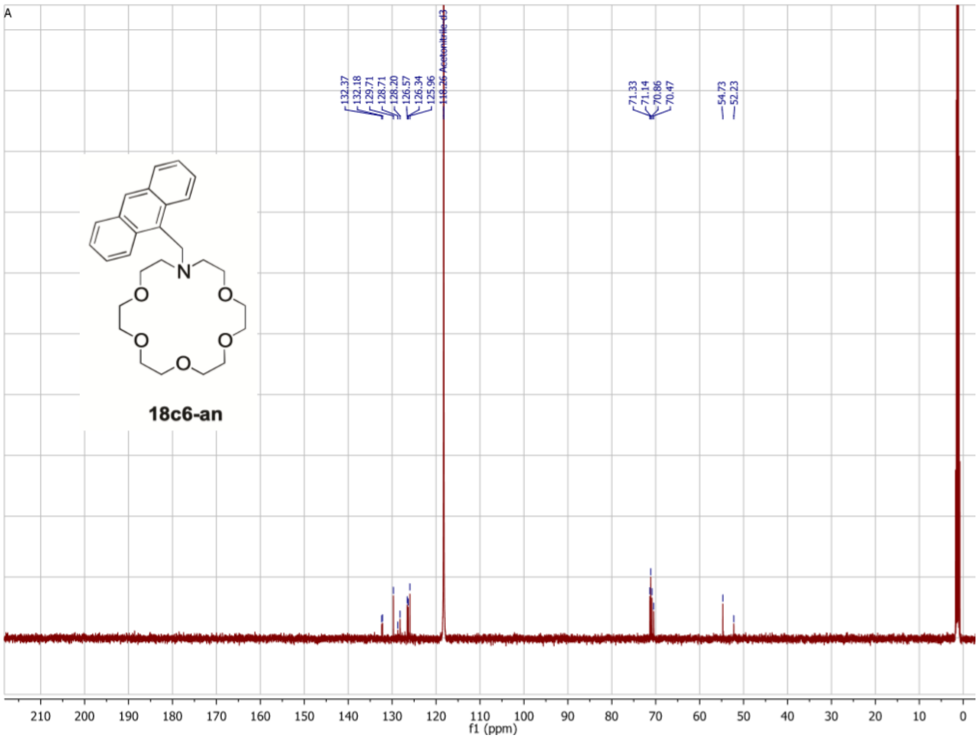

**Figure S8**: $^{1}$H NMR and $^{13}$C NMR of **18c6-an**

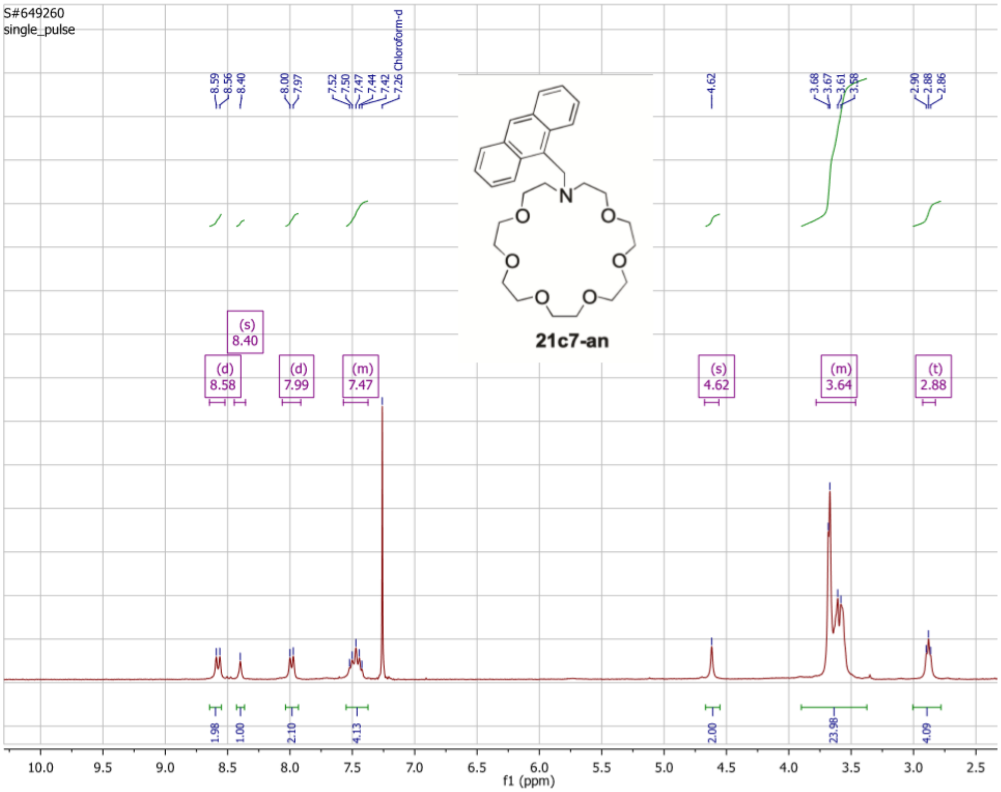


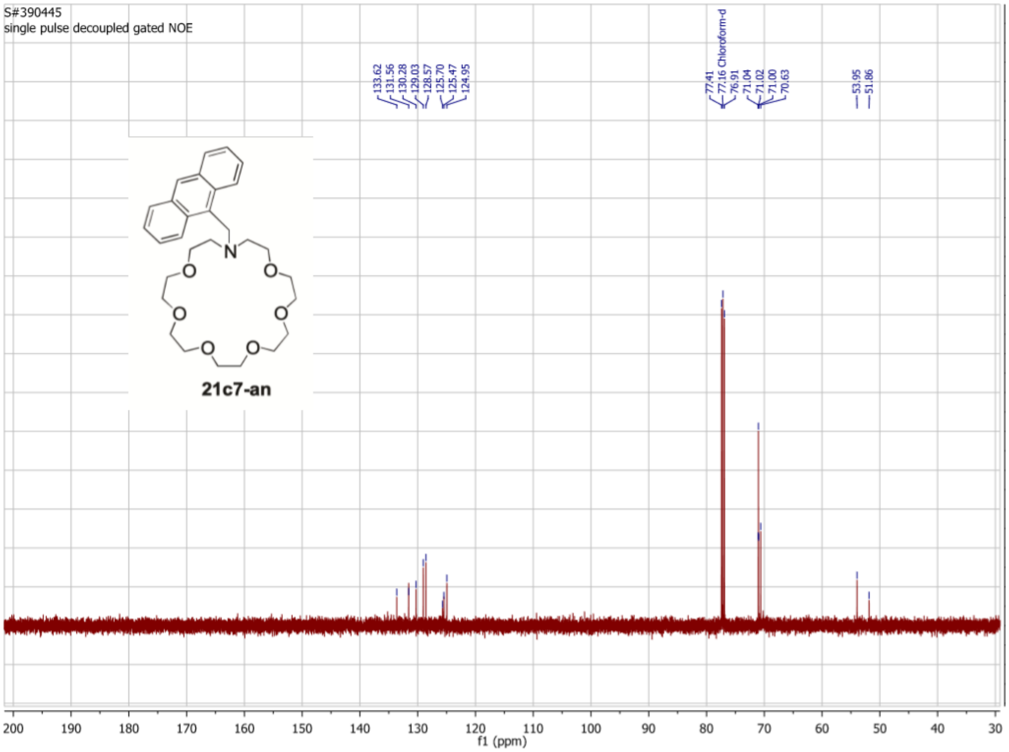


**Figure S9**: $^{1}$H NMR and $^{13}$C NMR of **21c7-an**

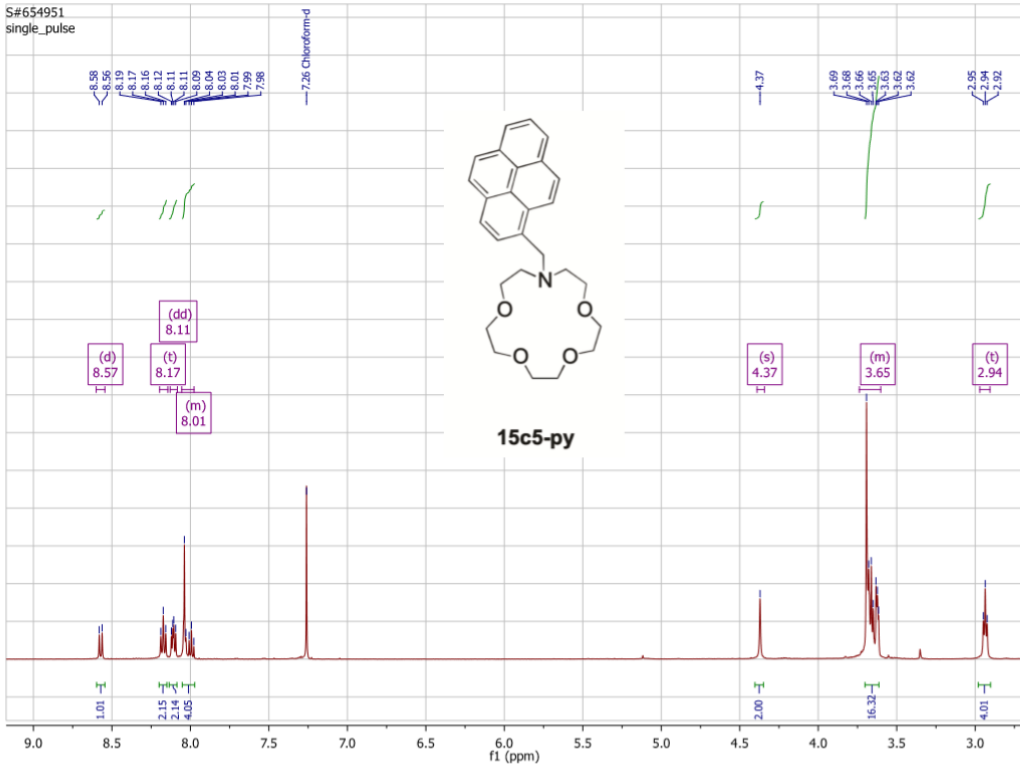


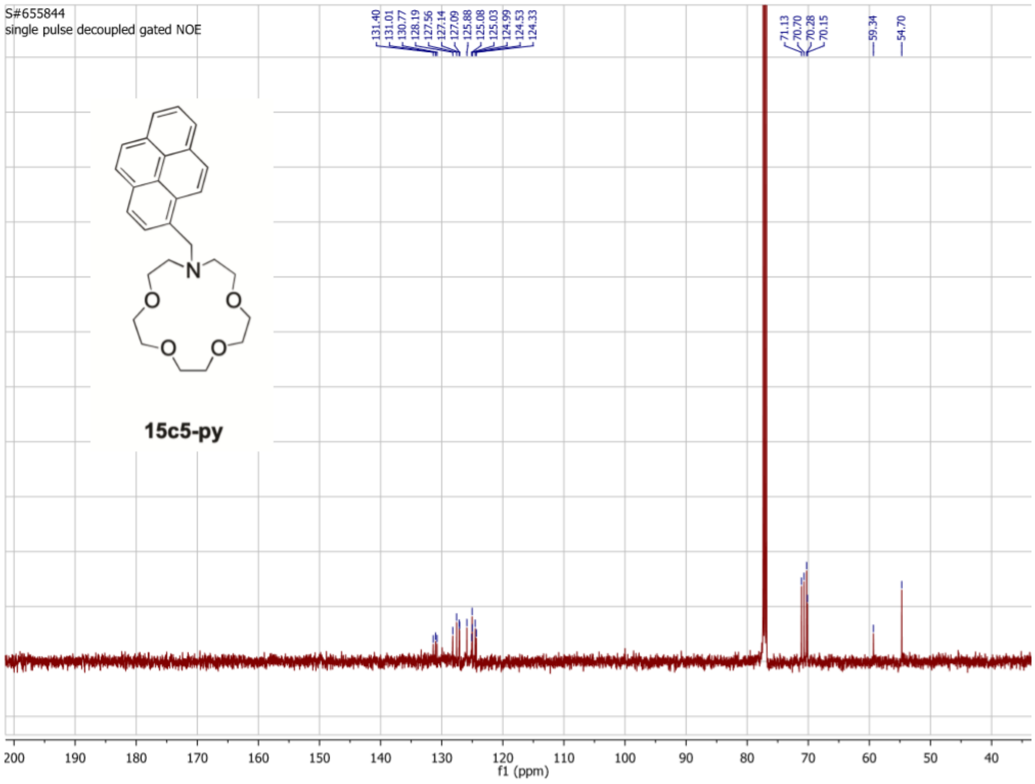


**Figure S10**: $^{1}$H NMR and $^{13}$C NMR of **15c5-py**

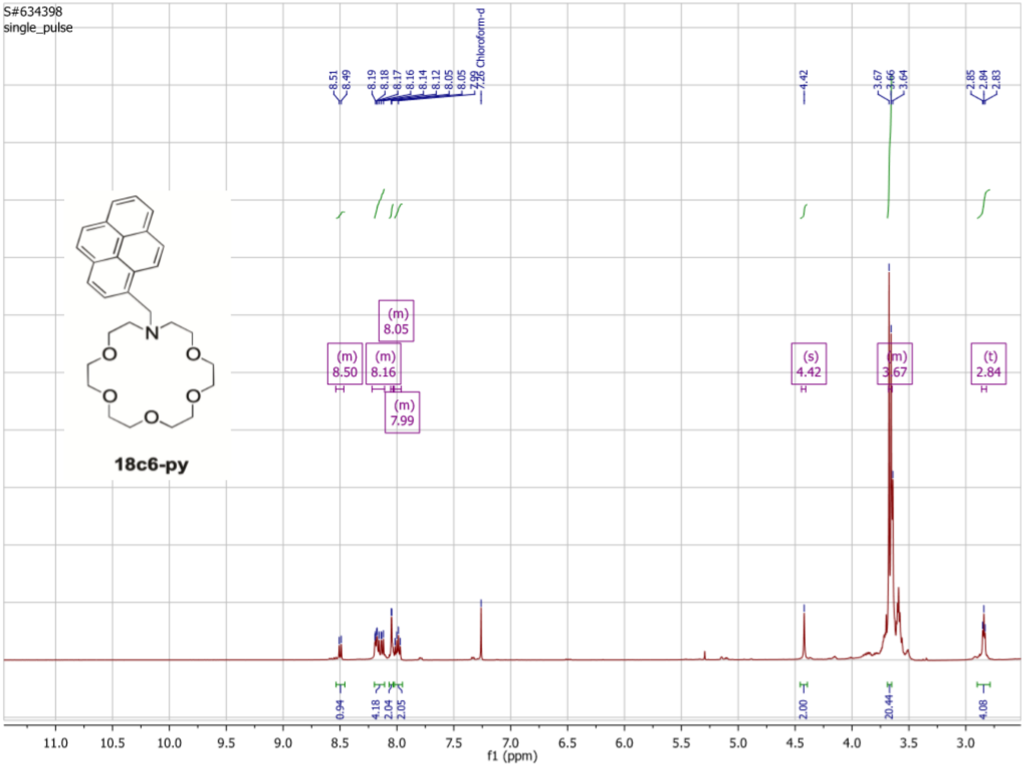


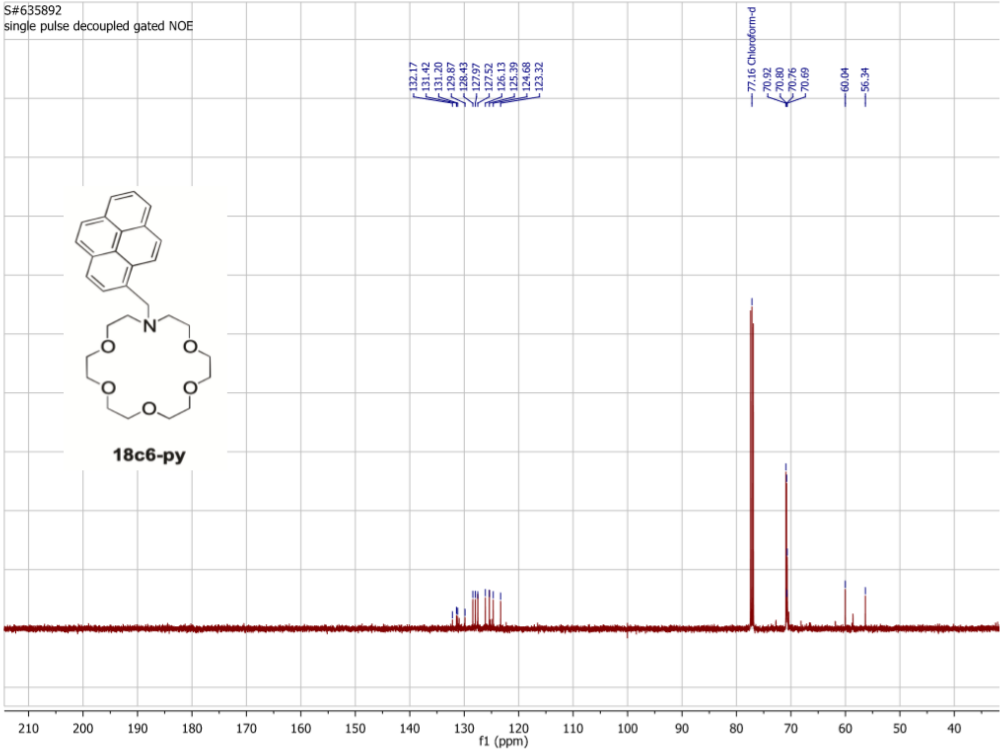


**Figure S11**: $^{1}$H NMR and $^{13}$C NMR of **18c6-py**

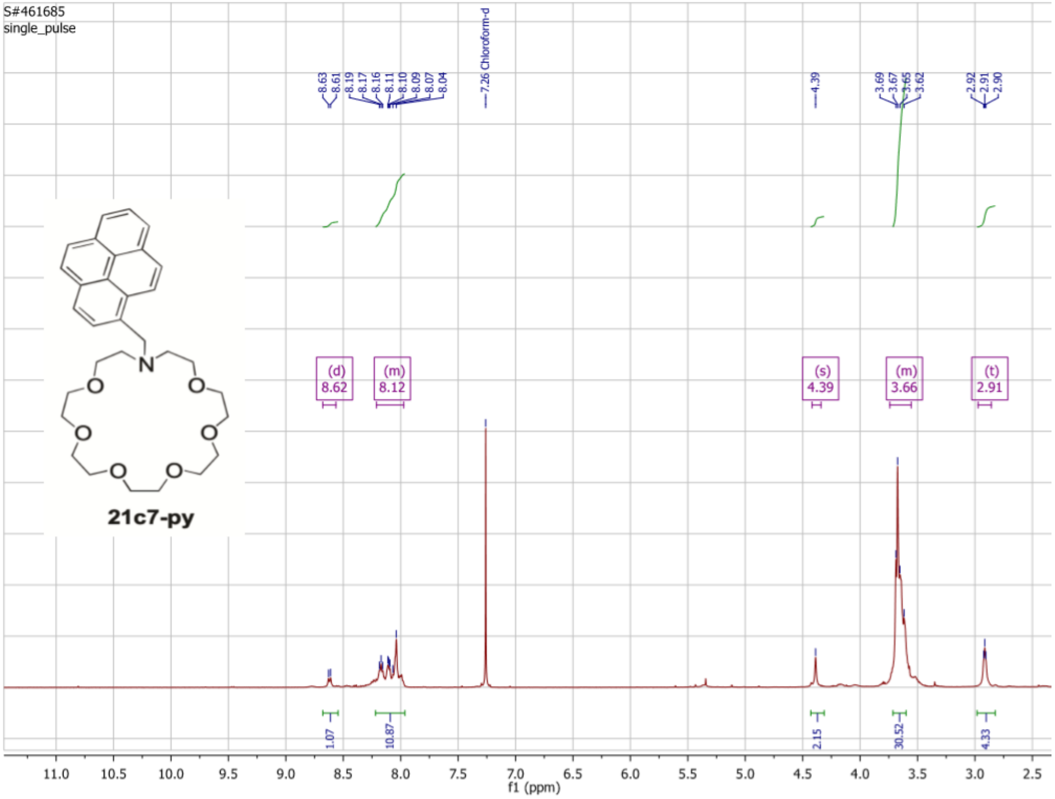


**Figure S12**: $^{1}$H NMR of **21c7-py**

**References:**


1. Bair, K. W. *et al*. 2-[(Arylmethyl)amino]-2-methyl-1,3-propanediol DNA intercalators. An examination of the effects of aromatic ring variation on antitumor activity and DNA binding. *J. Med. Chem*. **34**,1983-1990, DOI: 10.1021/jm00111a010 (1991).
2. Zeng, Z., Torriero, A. A. J., Bond, A. M. & Spiccia, M. Fluorescent and Electrochemical Sensing of Polyphosphate Nucleotides by Ferrocene Functionalised with Two $Zn^{II}$(TACN)(pyrene) Complexes. *Chem.- Eur. J.* **16**, 9154–9163. DOI: 10.1002/chem.201000882 (2010).
3. Malwal, S. R. & Chakrapani, H. Benzenesulfones as photochemically activated sulfur dioxide (SO2) donors. *Org. and Biomol. Chem*. **13**, 2399-2406. DOI: 10.1039/C4OB02466D (2015).
4. Zhan, W. *et al*. Integrating docking scores, interaction profiles and molecular descriptors to improve the accuracy of molecular docking: Toward the discovery of novel Akt1 inhibitors. *E. J. Med. Chem*. **75**, 11-20. DOI: 10.1016/j.ejmech.2014.01.019 (2014).
5. Luk;yanenko, N. G., Kulygina, E. Y., Bogashchenko, T. Y.& Yakovenko, I. S. Synthesis of monoazacrown ethers under phase-transfer catalysis. *Russ. J. Org. Chem.* **48**, 1345-1352. DOI: 10.1134/S1070428012100156 (2012).
6. Nakahara, Y., Kida, T., Nakatsuji, Y. & Akashi, M. New Fluorescence Method for the Determination of the Critical Micelle Concentration by Photosensitive Monoazacryptand Derivatives. *Langmuir* **21**, 6688-6695**.** DOI: 10.1021/la050206j (2005).
7. Mao, H.; Thorne, J. B.; Pharr, J. S.& Gawley, R. E. Effect of crown ether ring size on binding and fluorescence response to saxitoxin in anthracylmethyl monoazacrown ether chemosensors. *Can. J. Chem.* **84**, 1273-1279. DOI: 10.1139/v06-093 (2006).
